# Ten Simple Rules for organizing a non–real-time web conference

**DOI:** 10.1371/journal.pcbi.1007667

**Published:** 2020-03-26

**Authors:** Ana Arnal, Irene Epifanio, Pablo Gregori, Vicente Martínez

**Affiliations:** 1 Departament de Matemàtiques, Universitat Jaume I, Castellón de la Plana, Spain; 2 IMAC (Institut de Matemàtiques i Aplicacions de Castelló), Universitat Jaume I, Castellón de la Plana, Spain; 3 IF (Institut d'Estudis Feministes i de Gènere), Universitat Jaume I, Castellón de la Plana, Spain; Dassault Systemes BIOVIA, UNITED STATES

The present work describes the 100% virtual ATIDES (Avances en Tecnologías, Innovación y Desafíos de la Educación Superior) conference that was held between October 15 and 31, 2018, sponsored by Universitat Jaume I (UJI), Spain. Online conferences like this have been the subject of some controversy in the field of education over the last decade. Indeed, we have found a few texts that are against them. One of these is [1], whose authors claim that “interaction is not enough” to ensure efficient simulation of face-to-face contact. However, the Canadian academic community (for instance, the Centre for Distance Education at Athabasca University) is a strong advocate of online conferences (see [2,3]). Among other advantages, this kind of conference is “family-friendly,” i.e., they break barriers for researchers with family obligations [4], in particular many women [5]. In addition, these conferences overcome the drawback of parallel sessions at face-to-face conferences, at which participants must choose certain talks and miss others. Anderson and Anderson [6] even put forward environmental and economic arguments: “Transportation is a major contributor of carbon dioxide (CO2) emissions.” On the other hand, Abdullah [7] and Kear, Chetwynd, and Jefferis [8] look at the matter from another point of view that is also important: Social presence at online conferences.

Gichora and colleagues [9] propose 10 rules for organizing a virtual conference, but they only focus on real-time virtual conferences. Real-time virtual conferences emulate the structure of well-known on-site conferences, with the use of video-conferencing tools for connecting the participants in a virtual meeting. We, on the other hand, focus on non–real-time web conferences. In this kind of conference, a web platform connects the participants, but the event does not take place live. Communication can be made through posts on a forum, which can sometimes take hours or even days.

Non–real-time web conferences share many of the advantages of real-time virtual conferences, such as being family friendly and allowing the involvement of participants with a low budget for traveling, which makes this kind of conference more participative and inclusive. However, non–real-time web conferences have other advantages that real-time web conferences do not. Asynchronous interaction allows the attendees to have more time for reflection before they put forward their questions and comments, and, likewise, the speaker has enough time to prepare an appropriate answer. This makes up for the lack of face-to-face social interaction in this type of conference. In fact, numerous interactions occur via forums and are, in many cases, numerous and more informal and flexible than at classical scientific meetings [10]. Forums give rise to stimulating discussions like those that can occur at unconferences [11]. Furthermore, this asynchrony allows participants from all over the world to take part without worrying about time zone differences.

Another advantage is that it is not so greatly dependent on technology, unlike real-time web conferences: video-conferencing software is not necessary nor is a stable internet connection. In fact, we do not have to worry about the bandwidth like we do at real-time conferences. So, people in places that might not have very good or reliable internet connectivity can also participate.

Therefore, the cost of organizing a non–real-time web conferences is lower than for other scientific events. This means that the registration fees can be made very cheap (the registration fee for ATIDES 2018 was €35). This makes these conferences more affordable for everyone.

Furthermore, these conferences are more accessible since communications in non–real-time web conferences are text based. Although the conference can have an official language, language is not a barrier since Google Chrome can translate the text, so the content is accessible in any language without having to hire translation and interpreting services. Not only that, the content is also accessible for people with auditory disabilities.

In this paper, we describe the most notable data that have arisen from the event. Furthermore, we detail the steps taken previously during the planning stage as well as an evaluation of the results obtained. This conference was developed 100% virtually from beginning to end: The call for papers, the paper-selection process, the preparation and announcement of the proceedings, the process of presentations, discussions in forums, and issuance of the corresponding attendance and authorship certificates.

The following Ten Simple Rules are the result of the experience obtained from organizing the two editions of the 100% virtual ATIDES conference.

## Rule 1: Set up an organizing and scientific committee that is engaged with information and communications technologies

Effective committees are essential for the success of every kind of conference. In particular, a web conference also requires its organizers to have computer skills or, at least, have some knowledge of the computer tools that exist nowadays. As we will explain here, we had to handle web-hosting platforms and conference-management tools, and moreover, we created our own website. Therefore, an experienced organizing committee (supported by an expert information technology [IT] team, see Rule 3) makes this task easier. Regarding the scientific committee, its members should, at least, be aware of and somewhat familiar with information and communications technologies (ICTs), since they will have to interact by computer during the paper-selection process.

In our case, the ATIDES 2018 organizing committee was formed by researchers and teachers who were committed to the theme of the conference and willing to work as a team with ICT tools. They were also part of the scientific committee to ensure efficient coordination. The scientific committee consisted of 39 renowned researchers in teaching innovation from some of the universities listed in Table 1.

**Table 1:** Acronyms of participants’ institutions.

CNCIVIRTUAL: Universidad Centro Nacional de Capacitación Intensiva, Mexico.	ITSON: Instituto Tecnológico de Sonora, Mexico.
UAB: Universitat Autònoma de Barcelona, Spain.	UACO: Universidad Nacional de la Patagonia Austral, Argentina.
UANL: Universidad Autónoma de Nuevo León, México.	UAUSTRAL: Universidad Austral, Argentina.
UCAVILA: Universidad Católica de Ávila, Spain.	UCLM: Universidad de Castilla La Mancha, Spain.
UCM: Universidad Complutense de Madrid, Spain.	UCO: Universidad de Córdoba, Spain.
UCOMILLAS: Universidad de Comillas, Spain.	UHU: Universidad de Huelva, Spain.
UJI: Universitat Jaume I, Spain.	ULPGC: Universidad de Las Palmas de Gran Canaria, Spain.
UNAHUR: Universidad Nacional de Hurlingham, Argentina.	UNED: Universidad Nacional de Educación a Distancia, Spain.
UNEX: Universidad de Extremadura, Spain.	UNILEON: Universidad de León, Spain.
UNIOVI: Universidad de Oviedo, Spain.	UNIRIOJA: Universidad de La Rioja, Spain.
UNIZAR: Universidad de Zaragoza, Spain.	UNLP: Universidad Nacional de La Plata, Argentina.
UPM: Universidad Politécnica de Madrid, Spain.	UPOLI: Universidad Politécnica de Nicaragua, Nicaragua.
UPV: Universitat Politècnica de València Spain.	URJC: Universidad Rey Juan Carlos, Spain.
US: Universidad de Sevilla, Spain.	USAL: Universidad de Salamanca, Spain.
USFQ: Universidad San Francisco de Quito, Ecuador.	UTPL: Universidad Técnica Particular de Loja, Ecuador.
UV: Universitat de València, Spain.	UVG: Universidad del Valle de Guatemala, Guatemala.
UVIC: Universitat de Vic, Spain.	UVIGO: Universidad de Vigo, Spain.

## Rule 2: Correct timing of the conference

Exchanges in a non–real-time conference generally take much longer than in real-time conferences. This is particularly the case when participants belong to different time zones. Therefore, it is strongly recommended to establish a length of at least one or, preferably, two weeks for the whole conference. Moreover, it is very important to choose the right dates on which to hold the conference. Overlaps with similar conferences should be avoided to obtain maximum attention from potential participants. It is also necessary to consider periods that are not occupied by other activities (lectures, holidays, etc.). Nevertheless, even in the worst case, a non–real-time conference can fit in well with such other events thanks to the choice of a sufficiently long period.

The ATIDES conference took place during the last two weeks of October 2018, and it included participants mainly from Europe and South America, with a time difference of around eight hours. One week earlier, the inaugural speech was filmed. The institutional and academic authorities of UJI gave strong support for the event and showed a positive and warm attitude towards the participants.

At the very beginning of the conference, all attendees received a welcome message with a link to the virtual conference space. This message, which was sent to the general forum of the conference room, encouraged the participants to view the inaugural speech.

## Rule 3: Have an expert IT team to assist with web hosting and digital tasks

Efficient management of the ICT tools used is essential when carrying out an event like this. It is therefore necessary to have a team of expert technicians in this field who can help the organizers solve any technical problems that may occur as well as provide advice on the design and management of the virtual environment to be used. Online communication before, during, and after the conference (even in a non–real-time event) is the basis for effective interaction between all participants (organizers, speakers, and attendees), and this depends largely on the available computer support.

At the ATIDES 2018 conference, our IT team created the virtual conference space and had to react after a computer-server breakdown occurred during the course of the conference.

## Rule 4: Identify a good, visible hosting platform

Among the plethora of free and paid hosting services available, a clear and friendly website is absolutely necessary to encourage participation and increase the visibility of the conference. It is advisable to use a short and easy-to-remember web domain. The expert IT team can help with the best choice.

In our case, for the sake of greater freedom, affordability, and an elegant front end, we used a third-party free web design (https://www.weebly.com) for our first contact website.

## Rule 5: Develop the website and the virtual conference space

The website must contain all the usual key elements of a conference, such as "general information," "committees," "topics," "registration," and "access to the virtual conference space". In addition to the list of specific topics, it should also include keywords such as "web conference" and "asynchronous" (or "non–real-time") in order to ensure it is placed among the top internet search results of people seeking this kind of events.

Regarding the virtual conference space, it must be hosted on servers managed by the IT team. The IT team is responsible for installing, customizing, and maintaining a solid learning management system (LMS) program for managing the development of the conference. This software must be flexible and open, have a friendly interface, and facilitate asynchronous communication (interaction between participants can be promoted through open-forum spaces) and, optionally, synchronous communication (we offer a non–real-time event where real-time interaction is also welcome but not compulsory). The mature open-source Moodle or the more recent Blackboard Open LMS are two important examples. The constant availability of the servers is important (since authors and attendees may connect at any time of the day) but not as critical as for real-time web conferences. In the event of malfunction during the conference, the IT team must react quickly to restore connectivity.

We used our own space, which had been structured using a Moodle course to facilitate the development of the conference with a minimum hierarchical structure to organize participation in discussions.

## Rule 6: Have a good publicity campaign

Although publicity is important for any conference or event in general, in this case it can be considered a very important task to which we must pay special attention. The mechanism and development of non–real-time online conferences should be explained in the publicity campaign. In fact, a frequently asked questions (FAQ) section on the website is recommended.

Because our contact is exclusively online, the conference must be announced well in advance, and periodic calls should be made to encourage participation as speakers. The amount of notices and news should be greater than for a face-to-face conference. In this regard, it is important not to tire people with excessive information if they are not interested; they should be given the option to unsubscribe from the conference mailings.

Perhaps the place or the organizing institutions are little known, or, even if they are well known, it would offer added value to raise their profile and highlight the most attractive aspects.

Adequate diffusion of the accepted papers can help encourage participation. For example, indexing of the publication in the Conference Proceedings Citation Index by Clarivate Analytics could be requested.

In addition, in the call for papers it is important to be very clear regarding all details related to the format of the presentations, while submission deadlines must be perfectly explained on the website. As in face-to-face conferences, a call for papers was created, containing the purpose of the conference and the submission guidelines, together with important dates such as the submission deadline and the decision notification date.

## Rule 7: Specific actions for a virtual paper-selection process

Thirty years ago, works were sent by postal mail to be reviewed before being presented at a conference. Twenty years ago, these works were sent by email. But over the last decade, the use of web platforms for submission and peer review has been gaining popularity (see [12]). Thus, several conference-management tools have appeared, two of the most popular being the EasyChair conference system (see [13]) and the HotCRP conference-management system (see [14]). For more details, readers who are interested may consult Kanav, Lammich, and Popescu [15]. We cite, as alternative free platforms, ConfTool [16] and OpenConf [17]. Parallel to this, there has also been an increase in concern about verifying documentation submitted online in a confidential manner. Thus, for this new scenario the use of information security applications is necessary (see [18]). These tools are not exclusive to online conferences, but it is useful to mention them here since their use is necessary in virtual conferences.

In order to carry out the submission and review process considering the above requirements, we used the free version of the aforementioned EasyChair platform. This option allows us to handle all submissions with no restriction on the total size of uploaded files, except for a 20 Mb limit on the size of each individual file uploaded.

The platform allows us to set the parameters for the review process (e.g., reviewer assignations, reviewer forms for assessing papers, dates for opening the submission process, etc). The scientific committee, which includes all the reviewers, was entered on the platform, and invitation emails were automatically sent to all of them. Once the authors had submitted their work, two referees reviewed them. The referees assessed the contributions, and a subcommittee of the scientific committee made a decision and provided their comments and scores. The possible outcomes were rejected, resubmit with major changes, resubmit with minor changes, and accepted. The platform allows personalized emails to be sent to the authors or corresponding authors with the decision and comments from the reviewers. Those with the “resubmission” decision were given two weeks to make the changes indicated by the referees, and the subcommittee of the scientific committee then checked that those changes had been made. The format of the final accepted version was also reviewed in the EasyChair platform.

Although the scientific committee members are not required to be experts on ICTs, it is essential that they are active and willing to review the proposed papers in the aforementioned online platforms. In online conferences, papers can be more extensive than in face-to-face conferences. This is positive because papers can be more complete and detailed, but, on the other hand, the reviewers (in our case, the members of the scientific committee) have to make an extra effort.

A selection of the accepted papers for ATIDES 2018 was compiled in an electronic book with an international standard book number (ISBN). After a final review of the format of the selected publications, the proceedings book was edited with the help of our university editorial team. Moreover, although the papers were written in Spanish, we required the title, abstract, and keywords to be included in English too, since these proceedings were to be submitted for indexing in the Conference Proceedings Citation Index (Web of Science, Clarivate Analytics; see https://clarivate.com).

## Rule 8: Take actions to ensure the smooth running of the conference

Before starting the conference sessions, once the virtual conference environment is created, several necessary actions should be carried out to ensure that the conference runs smoothly. Most of all, it is important to create good quality and up-to-date content, both on the website and in the Moodle environment, to engage and continuously provide access to the audience.

We recommend regular communication with speakers whose contributions have been accepted and periodic announcements about the conference on general news forums and social media.

Although it is important to maintain constant communication to keep the participants interested in all kinds of conferences, in this case it is essential. In order to ensure a fluent participation of the attendees and authors and their exchanges and to avoid misunderstandings in discussions, we recommend the following actions be taken in the virtual conference space:

- Structure the conference space in clear blocks under the name of each topic within the conference.
- Create a separate forum for each contribution, named with the title of the paper and containing, as an attachment, the PDF document of the full text of the paper.
- Subscribe each author to his/her corresponding forum, and encourage him/her to post an introductory message on the opening day of the conference.
- Let the attendees choose their subscription to forums. Initially, they should not be subscribed to any forums, or they will receive many unwanted email notifications.
- Automatically subscribe each participant to a forum when he/she has posted a comment. In this way, he/she will receive an email notification when an author or other attendee posts an answer to his/her comment.
- Condition the certification of attendance to participation in a prescribed number of forum and/or posts.
- Condition the certification of authorship to authors of contributions whose forums have all participants' comments answered (by any of the authors).

One way to maintain interest and encourage participation is to propose the recording of short videos where the authors present their talks (following the rules explained in Lortie [19]). In the ATIDES conference, the video presentation of each paper had to have an image quality between 480 and 720 pixels and be accessible on YouTube. The PechaKucha [20] format is based on the fact that you have limited time to convey the fundamental idea of a proposal, since the recipient soon loses attention at an event where many speakers are involved. The idea is simple: 20 slides lasting 20 seconds each, i.e., a total duration of 6 minutes and 40 seconds. The authors who opted for this modality achieved a specific participation certificate.

## Rule 9: Make the content accessible in different languages

Many scientific events have only one official language, usually English (although in ATIDES 2018 the official language was Spanish). Hiring professional interpreters for simultaneous interpreting is not so common at scientific events. However, in non–real-time web conferences, machine translation can be exploited to increase the number of participants with different languages. The content of the website, papers, and forums is written, so the translation tool in Google Chrome can break down language barriers with a single click. Although Google’s take on an online interpreter will never be as good as a human translator, it can be a very good starting point.

As regards the videos of PechaKucha presentations, since they are on YouTube, its auto-caption feature can be used. Although YouTube’s automatic captions are not perfect and gender and dialect bias can be found [21], they can help to overcome language barriers.

Furthermore, it is possible to ensure website accessibility, for people with disabilities, people with slow internet connection, or users of different devices (e.g., from desktop or laptop computers to smartphones).

## Rule 10: Analyze data and obtain feedback to learn valuable lessons

All experiences must be evaluated to objectify reality and learn lessons that can be applied to future actions, which should be designed taking the available evidence into account. It is important to know and analyze the degree to which the initial objectives have been achieved so that we can design priorities and objectives that are consistent with reality. In our case, the indicators considered were the number of participants, the number of interventions in debates, the ease of handling the virtual environment, and the opinions of the actors involved during all stages of the conference (technicians, committees, participants, and speakers). Based on this evaluation, we may decide to continue with, modify, or discard the activity.

### Lessons learned from the ATIDES 2018 conference

Our non–real-time web conference, ATIDES 2018, included 242 participants and 56 papers signed by 110 authors. Fifteen of them gave a video presentation of their talk. While 80% of the papers led to at least 19 interventions, 50% of them exceeded 33 exchanges, and one of them even obtained 134 comments.

For the sake of clarity, we list the abbreviations or acronyms of the participating institutions in Table 1.

**Fig 1:**
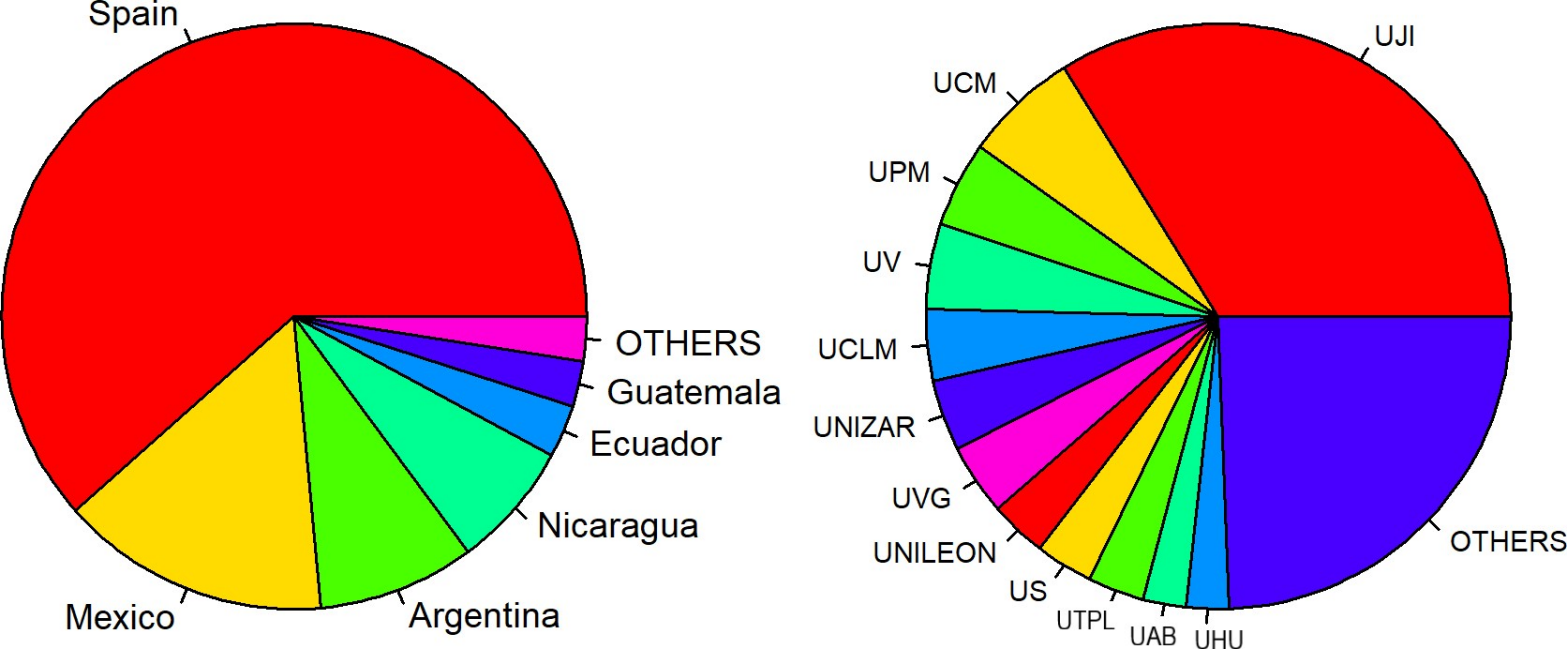
Left: Participants by country. OTHERS include participants from Venezuela, Brazil, Denmark, and South Africa. Right: Participants by institution. OTHERS includes UACO, UANL, UPOLI, UVIC, ITSON, UAUSTRAL, UCAVILA, UCO, UCOMILLAS, ULPGC, UNAHUR, UNED, UNEX, UNIOVI, UNIRIOJA, UNLP, UPCT, UPV, URJC, USAL, USFQ, and UVIGO. ITSON, Instituto Tecnológico de Sonora; UAB, Universitat Autònoma de Barcelona; UACO, Universidad Nacional de la Patagonia Austral; UANL, Universidad Autónoma de Nuevo León; UAUSTRAL, Universidad Austral; UCAVILA, Universidad Católica de Ávila; UCLM, Universidad de Castilla La Mancha; UCM, Universidad Complutense de Madrid; UCO, Universidad de Córdoba; UCOMILLAS, Universidad de Comillas; UHU, Universidad de Huelva; UJI, Universitat Jaume I; ULPGC, Universidad de Las Palmas de Gran Canaria; UNAHUR, Universidad Nacional de Hurlingham; UNED, Universidad Nacional de Educación a Distancia; UNEX, Universidad de Extremadura; UNILEON, Universidad de León; UNIOVI, Universidad de Oviedo; UNIRIOJA, Universidad de La Rioja; UNLP, Universidad Nacional de La Plata; UNIZAR, Universidad de Zaragoza; UPCT, Universidad Politécnica de Cartagena; UPM, Universidad Politécnica de Madrid; UPOLI, Universidad Politécnica de Nicaragua; UPV, Universitat Politècnica de València; URJC, Universidad Rey Juan Carlos; US, Universidad de Sevilla; USAL, Universidad de Salamanca; USFQ, Universidad San Francisco de Quito; UTPL, Universidad Técnica Particular de Loja; UV, Universitat de València; UVG, Universidad del Valle de Guatemala; UVIC, Universitat de Vic; UVIGO, Universidad de Vigo.

Fig 1 shows the details of the participants by country of origin (left) and affiliation (right).

**Fig 2:**
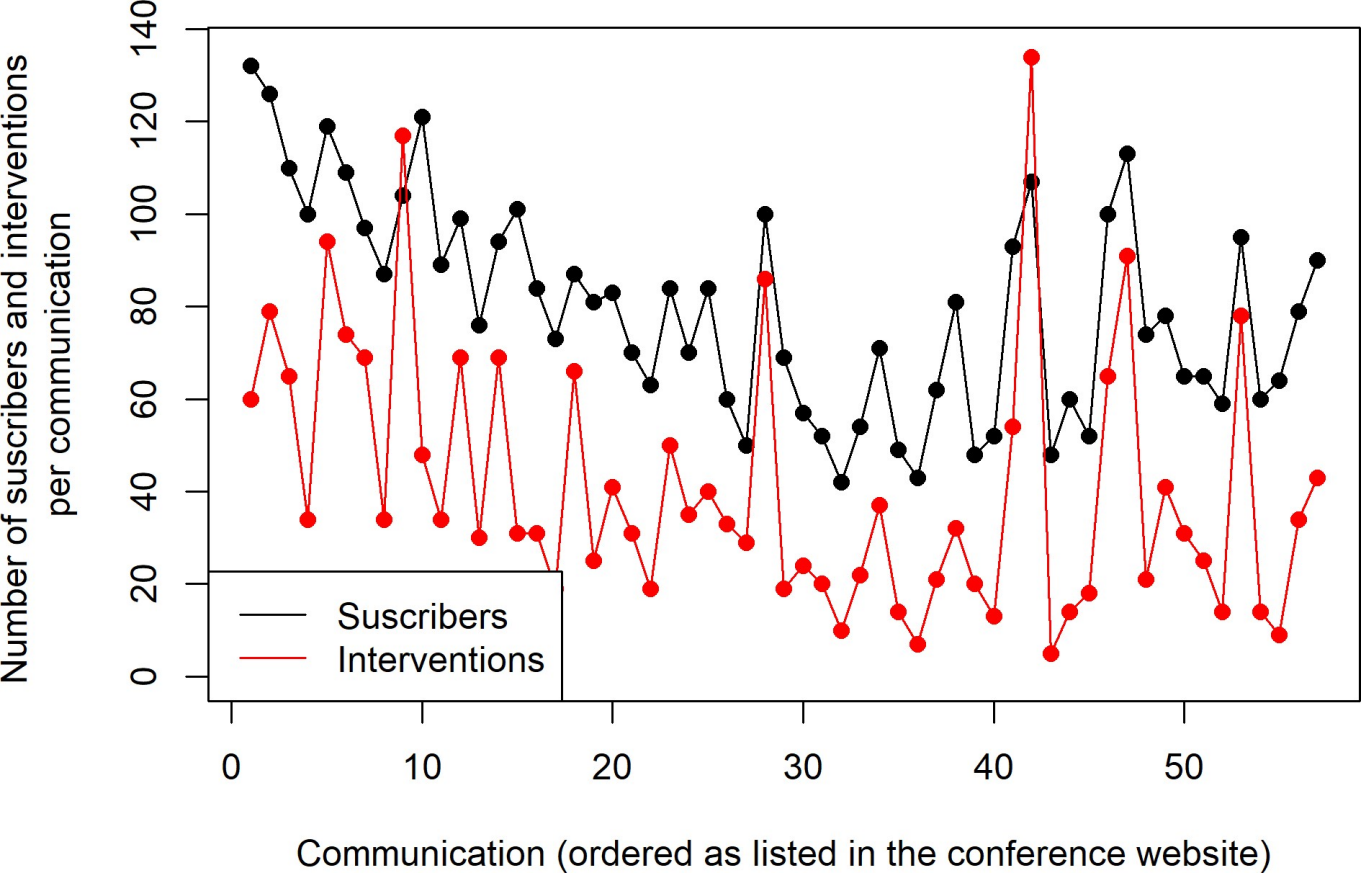
Participants subscribed to the debates and number of interventions per paper.

Fig 2 shows the number of participants who subscribed to (i.e., attended) the debate for a given paper and the number of interventions (questions, comments, etc.) in that particular debate. The data shown in this figure are surprisingly positive. It is rare to have more than 10 interventions in a face-to-face talk, but it was usual in this conference, with even more than 100 in some cases. This shows that in online conferences, asynchronous communication allows for deeper reflection when posing questions and also when answering them: Participants and authors can prepare their interventions more carefully.

**Fig 3:**
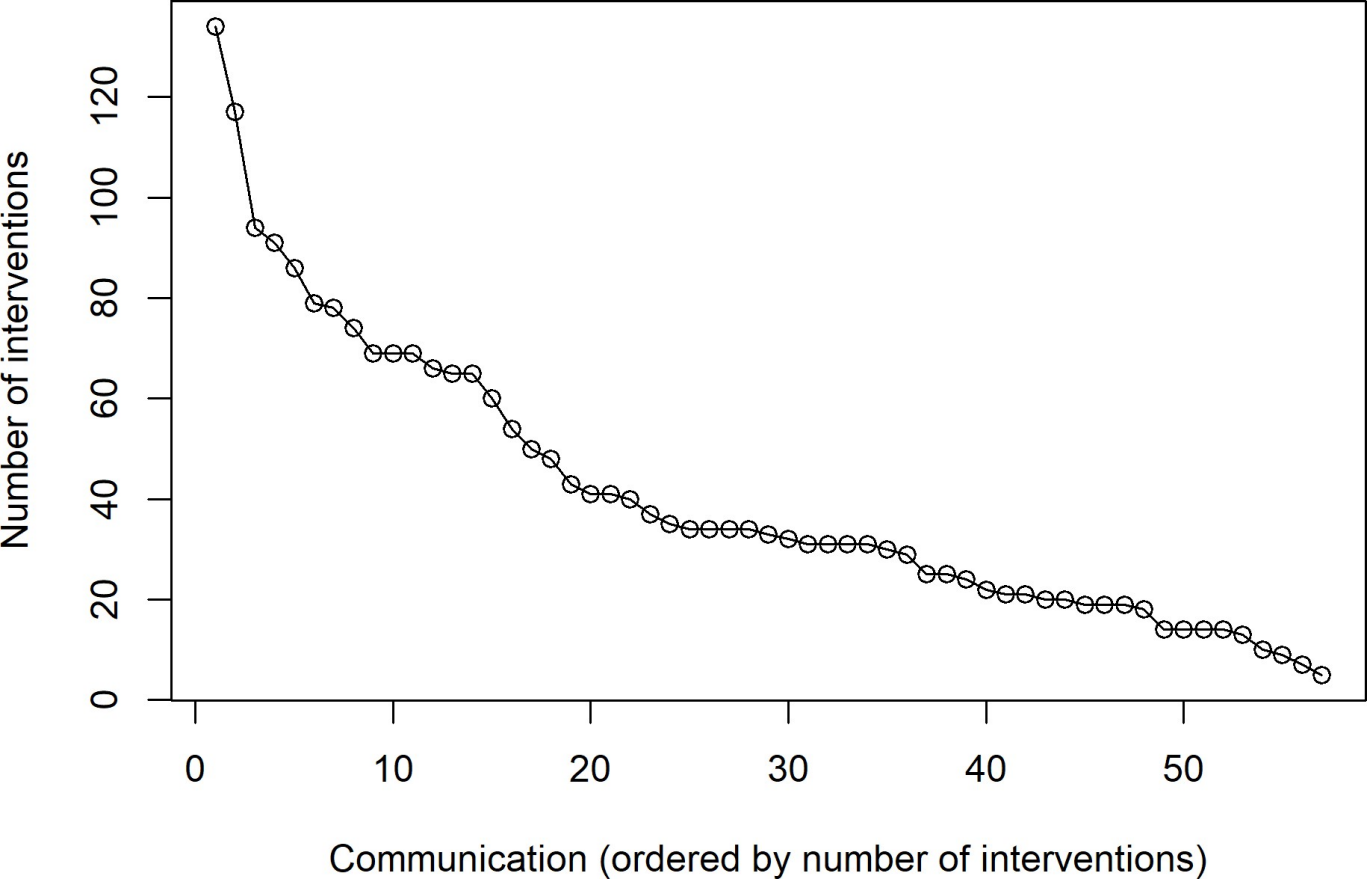
Papers ordered by number of interventions.

Fig 3 shows the papers ordered by number of interventions. It is clearly observed that the minimum number of interventions made in 80% of the presentations was 19, while the minimum number of interventions made in approximately half of the presentations was 33. These figures are difficult to achieve in face-to-face conferences.

**Fig 4:**
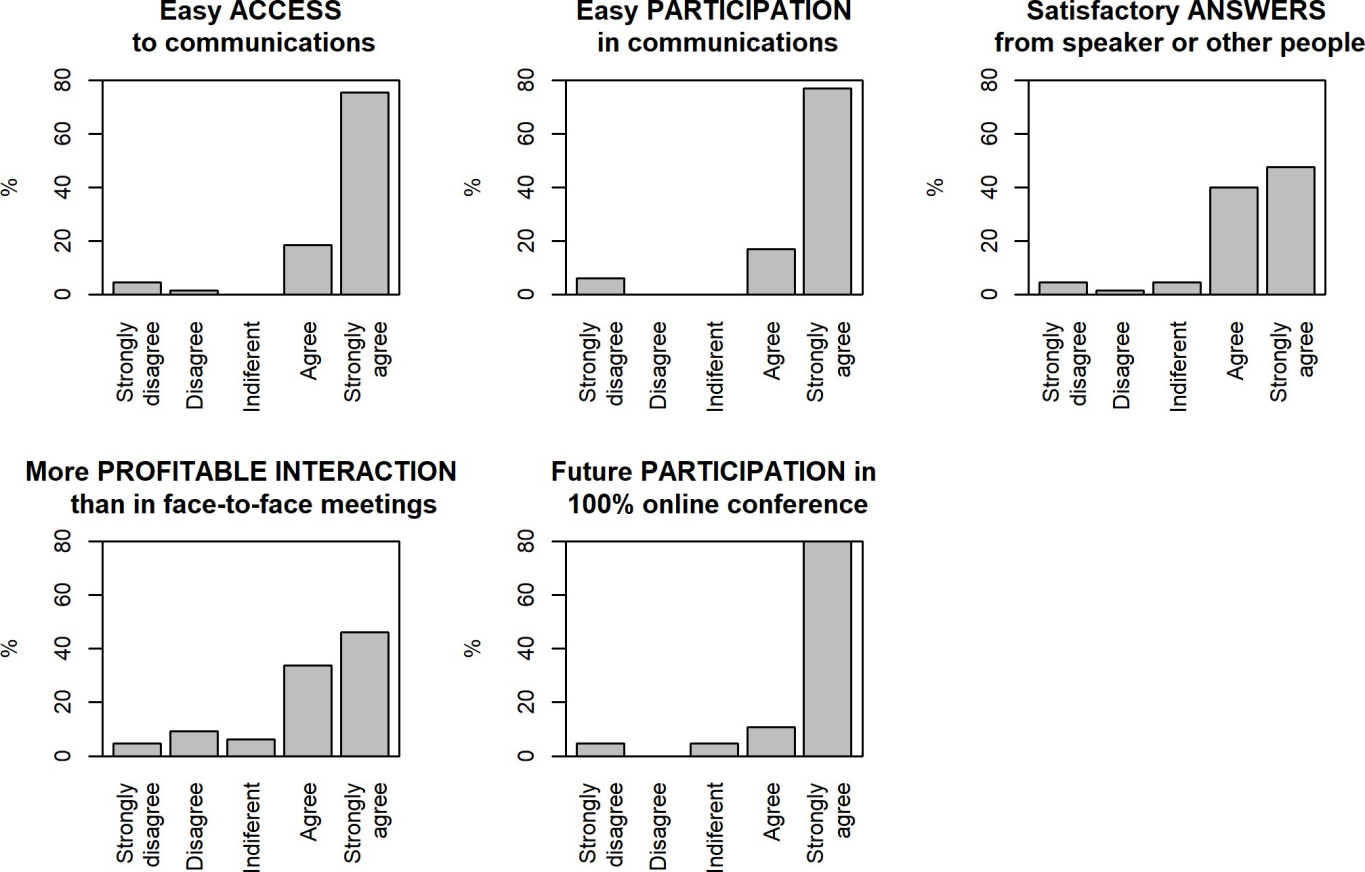
Attendee satisfaction results.

The attendee satisfaction data are shown in Fig 4. More than 90% of the attendees were satisfied or very satisfied with regard to access and ease of participation in the debates on the presentations and were willing to participate in future conferences of this type. In terms of satisfaction with the answers and their usefulness, the percentage was slightly higher than 80%, although this figure is still very positive.

**Fig 5:**
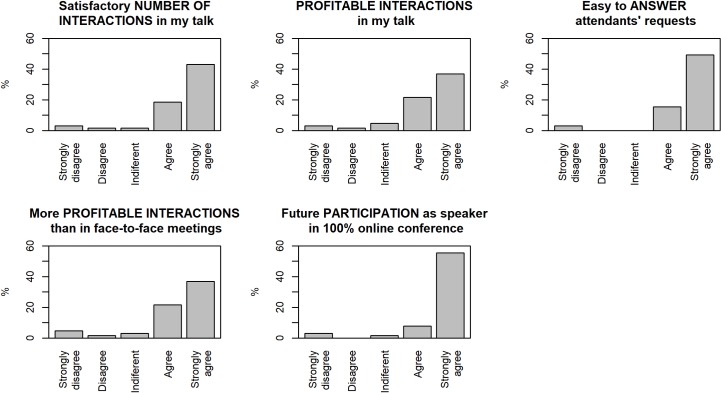
Speaker satisfaction results.

The speaker satisfaction data are shown in Fig 5. Approximately 60% of the speakers were satisfied or very satisfied with the number of interventions and their usefulness; this percentage exceeds 60% in terms of the ease of responding to the issues raised. More than 50% think that online participation is more profitable than face-to-face participation, and almost 60% will probably present a paper in future online conferences. Even though these data are positive, they indicate that there is still room for improvement in the preparation and development of online conferences. One aspect that needs to be improved is to promote closer, more personal interactions in a synchronous manner.

**Fig 6:**
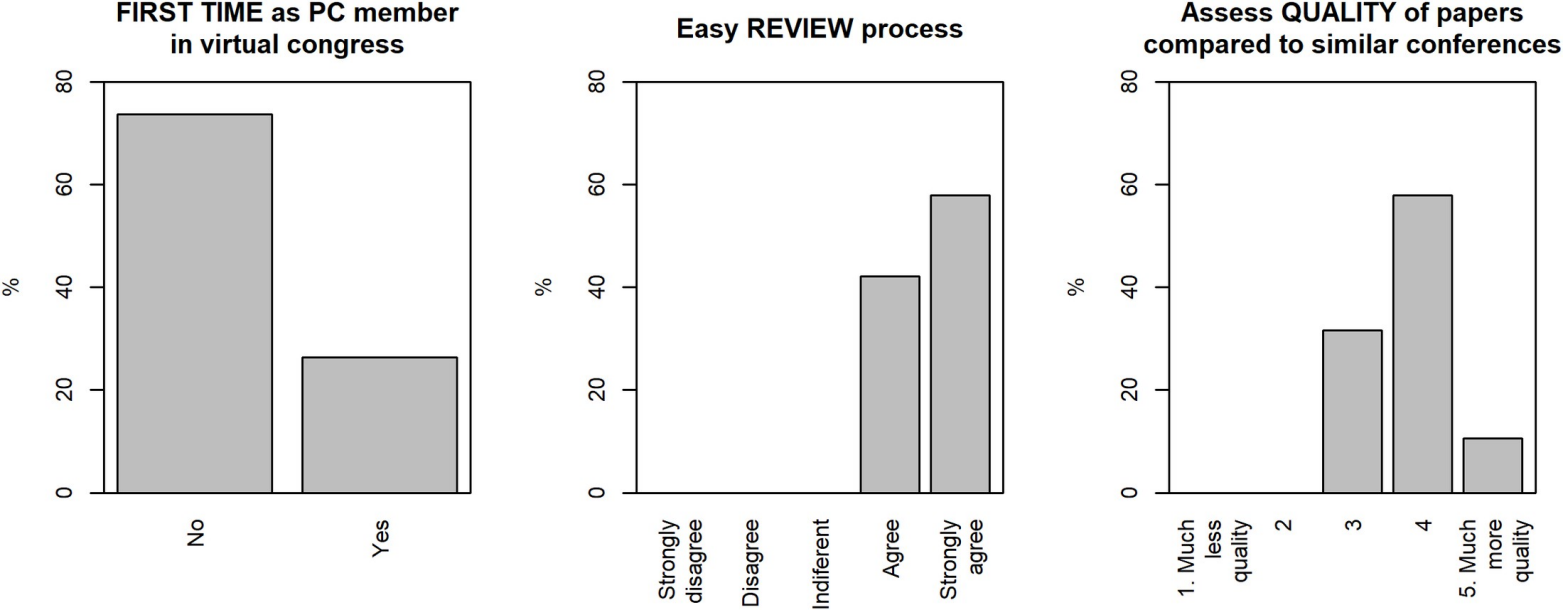
Scientific committee satisfaction results. PC, program committee.

Regarding the satisfaction of the scientific committee, it is worth mentioning that more than 70% stated (see Fig 6) that they had participated in virtual congresses before. This shows a substantial change in the trend of conference development and the enormous impact that it will have in the coming decade in terms of the use of ICT in the field of education. Nearly 100% of the respondents said they were satisfied or very satisfied with the review process. Regarding the quality of the papers, the committee considered that approximately 10% were very good quality and about 60% were good quality. Considering that one of the objectives of the conference was to be accessible to new researchers with few resources, the quality data can be considered satisfactory.

It is important to mention that online conferences are accessible to anyone who is interested in the subject. All that is needed to participate is an internet connection, so the participants’ geographical location does not matter. In addition, accommodation and travel expenses are not required, and the registration fees are very low. They are even family friendly and respectful to the environment.

Our proceedings were published by the Communication and Publications Service of UJI. This is an editorial member of the Union of Spanish University Publishers (UNE), which guarantees the dissemination and marketing of this work nationally and internationally.

The publication, with ISBN: 978-84-17429-54-6, can be found in volume 19 of the Educational Innovation collection (see [22]) and can be downloaded freely in print version and to electronic devices.

Although we initially proposed 10 thematic areas to the authors, in this edition the works were finally grouped into four main thematic areas:

- Virtual environments: distance education, e-Learning, b-Learning, massive open online course (MOOC), etc.
- Skills assessment and planning: skills assessment, improvement of quality, planning of the European Credit Transfer System (ECTS), gender, legal and economic aspects of education, etc.
- Innovative experiences in education: methodologies, content, assessment, etc.
- New technologies in education: videos, apps, tablets, telephony, social networks, blogs, etc.

Readers who are interested in educational innovation and new ICTs applied to education can also check the proceedings from the first and second editions of the conference, held in 2016 (see [23]) and 2018 (see [22]), respectively.

In summary, in view of the quantitative and qualitative results, the experience has been very positive and rewarding. The satisfaction surveys also show that it was well received.

The success of a virtual conference depends on many factors. One of the most important factors is the planning: Committee meetings, creation of the virtual environment, and announcements on forums and social media. In addition, the choice of an online conference-management system, such as EasyChair, speeds up the workflow and was satisfactory for authors and committee members. The structure of the conference website with a different forum for each talk provided quick and easy access to the discussions. Moreover, constant interaction between the participants increased their satisfaction.

Finally, as future work, we believe it is necessary to achieve better emulation of face-to-face contact in a synchronous way, as happens at face-to-face conferences. On this occasion, we launched a direct YouTube connection in the form of a coffee lounge, but the reality is that participation was limited.

We think that enhancing this connection through constant encouragement by a host in charge of this task throughout the conference could be effective.
